# Differences in risk factors for children with special health care needs (CSHCN) receiving needed specialty care by socioeconomic status

**DOI:** 10.1186/1471-2431-9-48

**Published:** 2009-07-31

**Authors:** Kristine A Lykens, Kimberly G Fulda, Sejong Bae, Karan P Singh

**Affiliations:** 1 Department of Health Management and Policy, University of North, Texas Health Science Center, School of Public Health, Forth Worth, TX, USA; 2 Department of Family Medicine, University of North Texas Health, Science Center, Primary Care Research Center, Fort Worth, TX, USA; 3 Department of Biostatistics, University of North Texas Health, Science Center, School of Public Health, Fort Worth, TX, USA

## Abstract

**Background:**

The purpose of this study is to identify factors affecting CSHCN's receiving needed specialty care among different socioeconomic levels. Previous literature has shown that Socioeconomic Status (SES) is a significant factor in CHSHCN receiving access to healthcare. Other literature has shown that factors of insurance, family size, race/ethnicity and sex also have effects on these children's receipt of care. However, this literature does not address whether other factors such as maternal education, geographic location, age, insurance type, severity of condition, or race/ethnicity have different effects on receiving needed specialty care for children in each SES level.

**Methods:**

Data were obtained from the National Survey of Children with Special Health Care Needs, 2000–2002. The study analyzed the survey which studies whether CHSCN who needed specialty care received it. The analysis included demographic characteristics, geographical location of household, severity of condition, and social factors. Multiple logistic regression models were constructed for SES levels defined by federal poverty level: < 199%; 200–299%; ≥ 300%.

**Results:**

For the poorest children (,199% FPL) being uninsured had a strong negative effect on receiving all needed specialty care. Being Hispanic was a protective factor. Having more than one adult in the household had a positive impact on receipt of needed specialty care but a larger number of children in the family had a negative impact. For the middle income group of children (200–299% of FPL severity of condition had a strong negative association with receipt of needed specialty care.

Children in highest income group (> 300% FPL) were positively impacted by living in the Midwest and were negatively impacted by the mother having only some college compared to a four-year degree.

**Conclusion:**

Factors affecting CSHCN receiving all needed specialty care differed among socioeconomic groups. These differences should be addressed in policy and practice. Future research should explore the CSHCN population by income groups to better serve this population

## Background

Most children are generally healthy requiring only primary and preventive health care. However a sub-group of children do have significant health problems. About nine percent of children were reported to currently have asthma, and five percent experienced one or more asthma attacks in the past year [1]. These children are among those likely to require specialty care in addition to primary and preventive care. Those with the most severe conditions are categorized as children with special health care needs. In 2005 parents reported that approximately eight percent of children had some type of limitation due to chronic conditions [1]. Also, in 2005, parents reported that 13.3% of children had taken prescription medication for at least three months, and over 15% of children over age five missed at least six days of school due to illness or injury [2].

The Maternal and Child Health Bureau defines children with special health care needs (CSHCN) as "those who have or are at increased risk for a chronic physical, developmental, behavioral, or emotional condition and who also require health and related services of a type or amount beyond that required by children generally"[3]. Approximately 13% of children in the United States met the Maternal and Child Health Bureau's definition of children with special health care needs (CSHCN) in 2001 – 2002 [4]. The prevalence is generally higher for older children, males, African Americans, and children in lower socioeconomic status (SES) households. Males are approximately 33% to 50% more likely to be CSHCN, and children in lower SES households are about 33% more likely [4,5]. Approximately 18% of CSHCN experience unmet needs for health care services, while 22% have difficulty obtaining needed referrals to a specialist [1].

### Specialty Care

Thirteen percent of children and adolescents 2 to 17 years of age are reported as having had a specialty care visit in the last year [6]. Non-white, poor, and uninsured children and adolescents are less likely to have seen a specialist [7]. Similar statistics apply to CSHCN, such that over 7% of CSHCN report an unmet need for specialty care. Older, poor, uninsured, and more severely ill or impaired CSHCN are more likely to have an unmet need for specialty care [8]. Additionally, maternal education of some high school and a high school diploma are also associated with a decreased odds of reporting a need for specialist care when compared to a four year college degree in one study using data from Texas children [9].

### Socioeconomic Status (SES)

SES is generally presented in the form of percent of federal poverty level (FPL), calculated as a combination of household income and the number of persons in a household. Disparities in income are substantial with more than one-third of Black (40%), Hispanic (35%), and Native American (41%) children in the United States living in households below the federal poverty level as compared to 10% of non-Hispanic white children [10].

Results of the National Health Interview Survey conducted in 1999 and 2000 provide insight into health disparities for adolescents 10 to 18 years of age based on SES. When compared to adolescents from households at ≥ 300% of the federal poverty level, adolescents in households at < 100 – 199% of FPL were significantly more likely to have fair or poor health, be limited in activity, and have a behavioral or emotional problem.

These children are also more likely to be uninsured, have no usual source of care when sick, have no personal health care provider, have had no visits to a health professional in the past year, and be unable to get medical care due to costs. Most of these trends continued even into households at 200 – 299% of FPL [11].

Similar trends in health status and health care access occur in CSHCN from different SES levels. Parents or guardians of CSHCN belonging to households < 199% FPL are more likely to report not having received all needed specialty care [8]. CSHCN in households < 100% of FPL and 100 – 200% of FPL are greater than four times more likely to not have insurance when compared to households ≥ 200% of FPL [12]. Van Dyck, Kogan, McPherson et al. [4] also demonstrate that when compared to households ≥ 400% of FPL, CSCHN in households at 0 – 399% of the FPL are more likely to have unmet needs for care.

### Study Objective

Our study examined whether CSHCNs in families at different income levels had similar or different factors that affected their receiving needed specialty care. The factors examined included demographic characteristics, geographic location, insurance status, and severity of their condition.

## Methods

### A Conceptual Framework for SES Status and Access to Specialty Care

The "system" to address health care needs of CSHCNs evolved over the past four decades, in the U.S., through implementation of a number of categorical programs, such as those administered by the Maternal and Child Health Bureau, and entitlement programs, mainly Medicaid. Each of these programs created rules to determine eligibility for services. These rules were largely based on family income and age of child. Services and provider networks were independently established for these programs. In the late 1990s the SCHIP legislation increased eligibility for public insurance, allowing states to expand Medicaid eligibility, establish free-standing programs, or both. The evolution of these policies to address children's health care needs has led to CSHCNs being served by this entire spectrum of programs and often different provider networks.

For our study we examined three income groups. The first group of these children are in families with incomes < 200% of the Federal Poverty Level (FPL). These children are primarily served by public programs and insurers. The second group of children are in families between 200–299% of FPL. Their access to specialty care varies considerably. The Medicaid and SCHIP programs in most states have income eligibility limted to < 200% of FPL. However several more progressive states have expanded eligibility to 250% or even 300% of FPL[13]. Thus the likelihood of being uninsured or publically or private insured varies by state for this income group. These income thresholds also vary by age across the states for Medicaid eligibility for age groupings of infants, ages 1–5, ages 6–16, and ages 17–18. [13]. The highest income group 300% of FPL and higher are very predominantly served through private insurers and providers.

Consequently the availability of specific services and access to providers became dependent on the SES level of the child's family. Thus examination of access to care for CSHCN is best viewed through a lens that separately identifies SES level. The impact of other factors such as age, severity of condition, income, region of residence, and family structure may be quite different for children in families with different income levels. Thus the focus of our study is to examine what are these differences and what do they mean for policy and practice regarding Children with Special Health Care Needs.

### Data

All data were obtained from the National Survey of Children with Special Health Care Needs (CSHCN) conducted from October 17, 2000 through April 30, 2002. The National Survey of CSHCN was developed by the Maternal and Child Health Bureau (MCHB) to monitor the health care of CSHCN at the national and state level [13]. The survey was conducted in conjunction with the Centers for Disease Control and Prevention's (CDC), National Center for Health Statistics using the State and Local Area Integrated Telephone Survey (SLAITS)[14].

A total of 196,888 potential participants were screened according to the CSHCN criteria, and data were collected for 38,866 CSHCN. Because of the comprehensive administration of the National Survey of CSHCN, there were not substantial missing data. Therefore, missing data were excluded from the analyses. Study procedures for this analysis were approved by the Institutional Review Board at the University of North Texas Health Science Center with exempt review.

### Statistical Analysis

All analyses were conducted using SAS version 9.1.2 to adjust for the complex survey sample design.

CSHCN were defined as needing specialty care if a response of yes was provided to the following question: "During the past 12 months, was there any time when (CHILD) needed care from a specialty doctor?" Having received all needed specialty care was determined using the following question: "Did (CHILD) receive all the care from a specialty doctor that {he/she} needed?"

Descriptive statistics are provided for having received all needed specialty care, age, gender, race (white only, black only, other), ethnicity (Hispanic, non-Hispanic), maternal education, health insurance status (Medicaid, private, SCHIP, other, uninsured), severity of condition, relationship of respondent to CSHCN (mother, other), number of children living in the household, number of adults living in the household, and geographical location of the household (Midwest, Northeast, South, West). As illustrated in Additional file 1, significant differences among SES strata were identified for all demographic characteristics except gender using chi-square analysis and ANOVA, as appropriate. This confirms the importance of stratifying the analysis by SES levels. All analyses were conducted using SAS version 9.1.2 to adjust for the survey sample design.

Multiple logistic regression was conducted. All independent variables were included in the multiple logistic regression models, and the models are estimated for each income stratum with identical variables presented for each SES stratum. Odds ratios and 95% confidence intervals as well as standardized beta coefficients and 95% confidence intervals are presented for each variable. Standardized beta coefficients have been used to compare independent variables among each SES stratum. The – 2 log likelihood is presented to illustrate the fit of each model.

Interaction terms were included in the original model for each SES stratum [15]. All interaction terms were tested for colinearity before inclusion in the models. Pearson correlation coefficients ranged from -0.11 to 0.11, except for private insurance * Medicaid (*r *= 0.61). Interactions were tested in the regressions. Few interaction effects were found to be significant. Therefore interaction results are not reported.

## Results

There were 38,866 children with special health care needs (CSHCN) represented in the National Survey of Children with Special Health Care Needs, 2000 – 2002. The distribution of each variable above, except gender, was statistically significant between SES levels at p < 0.01. Descriptive statistics for each variable by SES are provided in Additional file 1. Of the CSHCN in the sample 20,472 (53%) were noted to have a need for specialty services and are included in the analysis. The raw numbers reported are unweighted sample counts. The percentages in parentheses are weighted population proportions. Of the CSHCN with a reported need for care from a specialist in the past year, 19,270 (92.75%) reported having received all needed care. The proportions are successively larger for each income stratum: 5,881 (86.57%) in the < 199% of FPL; 3,488 (94.13%) in the 200 – 299% of FPL; and 8,128 (96.18%) in the ≥ 300% of FPL. These results demonstrate that as the SES status of the CSHCN is positively associated with the likelihood of receipt of all needed care from a specialist.

### Comparison of Regression Models

Multiple logistic regression was conducted. All independent variables were included in the multiple logistic regression models, and the models are estimated for each income stratum with identical variables presented for each SES stratum. Odds ratios and95% confidence intervals as well as standardized beta coefficients and 95% confidence intervals are presented for each variable. Standardized beta coefficients have been used to compare independent variables among each SES stratum. The – 2 log likelihood is presented to illustrate the fit of each model.

Interaction terms were included in the original model for each SES stratum [15]. All interaction terms were tested for colinearity before inclusion in the models. Pearson correlation coefficients ranged from -0.11 to 0.11, except for private insurance * Medicaid (*r *= 0.61). Interactions were tested in the regressions. Few interaction effects were found to be significant. Therefore interaction results are not reported.

### Comparison of Regression Models

Age was only significant in the ≥ 300% of FPL stratum, suggesting that older children were more likely to have received all needed specialist care. Although age was significant for this group the odds ratio of l.07 indicates that this is a minor difference. Severity of condition was significant only for the 200–299% FPL stratum. For this group of children more severe conditions were less likely to receive needed specialty care. Gender and race were not found to be significantly associated with receipt of needed specialty care. Hispanic children were found to be more likely to receive needed specialty care. However, this association was only significant for the lowest economic stratum. Maternal education was not found to have a significant impact on children's receipt of need specialty care in the lower two income strata. For children in the 300% and greater FPL group mother's education of less than a college degree was negatively associated with the likelihood of receiving needed specialty care. However, this association was only significant for those with a high school degree and some college.

Compared to privately insurance having Medicaid or SCHIP insurance coverage was not found to significantly impact the likelihood of children's receipt of needed specialty care. A child having no insurance is negatively associated with receipt of needed specialty care. These associations were not statistically significant at the .05 level. However for children in the lowest and highest income groups this negative associate was significant at the .10 level.

Region of residence for these children was found to have some impact. The only significant association was found for children in the highest income group residing in the Midwest where the association was positive compared to the Northeast suggesting higher income children in the Midwest are more likely to receive needed specialty care.

Family structure did have an impact on children's receipt of needed specialty care. Having additional adults in the household was positively associated with children's receipt of specialty care. This association was very strong for children in the lowest income households and was significant only for this income group. Conversely, the number of children in the household is negatively associated with children's receipt of needed specialty care. This impact is also strongest for the lowest income group and only significant for them.

The -2 Log Likehood, a measure of the extent to which the model is consistent with the data, illustrated that the < 199% of FPL stratum was the best fit model. The middle income stratum model was the worst fit.

## Discussion

Our study found that 7.25% of CSHCN who needed care from a specialist did not receive it. This exactly matches the study by Mayer et al. (2004) [8] who also analyzed data from the National Survey of Children with Special Health Care Needs, 2000 – 2002. The current research, however, differs from that of Mayer et al. by examining each of three SES strata independently and then comparing how various risk factors affect each stratum. Mayer et al. (2004) included three levels of SES (< 100% of FPL, 100 – 199% of FPL, and ≥ 200% of FPL) as a factor in the model, thus, controlling for SES.

The current analysis revealed that age is a significant risk factor of having received all needed care from a specialist, but only in the ≥ 300% of FPL stratum. Older children were more likely to have received needed specialty care. One possible explanation is that the disability or need for extra care may not be apparent until the child is older and either enters school or is observed in group settings with other children their age. However, the positive odd ratio for age was relatively small for this group.

The severity of the condition (0 – 10 scale) only demonstrated a significant effect on receiving all needed care from a specialist in the 200 – 299% of FPL stratum, demonstrating that as the severity of the condition increased, the likelihood of having received all needed care decreased.

Our study did find a significant association for ethnicity. When stratified by SES a significant association for Hispanic children was discovered in the < 133% of FPL stratum. The association was positive but not significant for the higher income groups. Our finding of a relationship may be explained by differences in perceived needs and expectations of providers between Hispanic and non-Hispanic parents of CSHCN [16]. Further research into how these expectations may differ with SES would help explain this interesting finding.

Maternal education significantly affected having received all needed care from a specialist in the 200 – 299% of FPL and ≥ 300% of FPL strata. For CSHCN in the 200 – 299% of FPL stratum, a maternal education of less than or equal to a high school diploma or GED increased the likelihood of having received all needed care as compared to having a four year college degree or more. This is reverse from what would be expected and what was found in the ≥ 300% of FPL stratum. In the highest SES stratum, a lower maternal education was negatively associated with having received all needed care. One explanation for this finding could be that women in the lower SES stratum who are also less educated may have less knowledge of which specialty services could benefit their special needs child and perhaps a lower expectation of the health care system. An appropriate response to this finding would be to increase the efforts to properly educate these mothers regarding the benefits of specialty care. This association, though, was only significant for having some college, but not a four year college degree. The importance of maternal education was not identified when the analysis was not stratified by SES.

Being uninsured and type of health insurance are generally considered factors that affect unmet health care needs for CSHCN. In our study, this association did not present across all SES strata. Being uninsured only significantly predicted having received all needed care in the 133 – 199% of FPL stratum. No significant differences were found for publically and privately insured children. Previous research from the National Survey of Children with Special Health Care Needs, 2000 – 2001 demonstrated that CSHCN who did not meet 3 health insurance components of coverage, continuity, and adequacy demonstrated a greater than three times odds of having one or more unmet health care needs [17]. Mayer et al. (2004) also found that CSHCN who were uninsured at some point in the past 12 months less likely to have received all needed care from a specialist.

The geographical location of the household played a small role in having received all needed care from a specialist, particularly in the highest income group. For these children living in the Midwest was positively associated with receiving specialty care and the association was significant. Regional differences in availability of specialists to uninsured and Medicaid vs. other public and privately insured children is an issue which warrants further investigation[18].

Family structure is important for Children with Special Health Care Needs. The number of adults in the household was positive and significant in the lowest income group. As the number of adults in the household increased, the likelihood of having received all needed care increased. It is especially important in the lower SES strata to have family support with providing care for CSHCN. These poorer families may have additional financial barriers to accessing specialty care such as finding affordable child care for other children to facilitate specialty appointments [19]. The number of kids in the household significantly predicted having received all needed care in the < 199% of FPL stratum. The coefficients for all income groups were negative, suggesting that the number of kids in the households reduced the likelihood of receiving needed specialty care in poor families. One possible explanation is that when there are more children, there is less time to dedicate to the needs of the CSHCN and the barrier mentioned above regarding child care.

## Conclusion

Our results show that the income of the families in which CSCHN reside have an important effect on which factors contribute to the probability that these children receive all needed specialty care. Financial barriers in addition to health insurance deserve additional research and policy focus. Some of these barriers could be addressed through practice by providing for child care of other children during specialty appointments through onsite or subsidized arrangements for poorer children. State policy regarding reimbursement levels and requirements for specialists on Medicaid and SCHIP provider networks could address the geographical disparities of specialty care access.

Both referring and specialty physicians should be attentive to the differences our study found in access to specialty care between income groups as should programs that serve different groups of CSCHN. Lower income children have significant barriers to access to specialty care, beyond insurance, which need to be addressed. Further research should incorporate SES differences into the research design. Also policymakers and practitioners can take these differences into consideration in planning for services to these children.

## Competing interests

The authors declare that they have no competing interests.

## Authors' contributions

KL is the primary author of the manuscript. She participated in the research design, supervised the analysis, and contributed to the results and discussion. KF participated in the research design, conducted the data analysis, contributed to the writing and editing of the manuscript. KS contributed to the research design and discussion sections. SB contributed to the research design and discussion sections. These authors are the sole contributors to this project. All authors read and approved the final manuscript.

## Pre-publication history

The pre-publication history for this paper can be accessed here:



## Supplementary Material

Additional File 1 Table 1 Overall Demographic Characteristics; Table 2 Demographics by SES Stratum and Access to Specialty Care; Table 3 Mulitple Logistic Regression for Each SES Stratum. Click here for file
