# Supplementary material for: Differences in risk factors for children with special health care needs (CSHCN) receiving needed specialty care by socioeconomic status
Source: BMC Pediatr. 2009 Jul 31;9:48. doi: 10.1186/1471-2431-9-48 (PMC2729735; doi:10.1186/1471-2431-9-48)
Supplement: Additional File 1 — Table 1 Overall Demographic Characteristics; Table 2 Demographics by SES Stratum and Access to Specialty Care; Table 3 Mulitple Logistic Regression for Each SES Stratum. [file 1471-2431-9-48-S1.doc]

| **Table 1. Overall Demographic Characteristics** |  |  |  |  |  |  |  |  |  |
| --- | --- | --- | --- | --- | --- | --- | --- | --- | --- |
|  | **Total** | **< 133%** | **133 – 199%** | **200 – 299%** | **= 300%** | **Overall** |  |  |  |
|  |  | **of FPL** | **of FPL** | **of FPL** | **of FPL** | **p** |  |  |  |
|  | **n (%)** | **n (%)** | **n (%)** | **n (%)** | **n (%)** |  |  |  |  |
| **Specialty Care** |  |  |  |  |  | < 0.01 |  |  |  |
| Yes | 19,270 (92.75) | 3,310 (84.26) | 2,571 (89.90) | 3,488 (94.13) | 8,128 (96.18) |  |  |  |  |
| No | 1,202 (7.25 | 420 (15.74) | 240 (10.10) | 199 (5.87) | 267 (3.82) |  |  |  |  |
| **Insurance** |  |  |  |  |  |  |  |  |  |
| Medicaid |  |  |  |  |  | < 0.01 |  |  |  |
| Yes | 9,686 (25.82) | 5,296 (68.31) | 1,715 (34.38) | 975 (15.14) | 741 (5.17) |  |  |  |  |
| No | 29,180 (74.78) | 2,546 (31.69) | 3,793 (65.62) | 6,045 (84.86) | 14,118 (94.83) |  |  |  |  |
| Private |  |  |  |  |  | < 0.01 |  |  |  |
| Yes | 28,489 (72.11) | 2,312 (27.73) | 3,540 (60.74) | 5,903 (83.35) | 14,150 (94.32) |  |  |  |  |
| No | 10,377 (27.89) | 5,530 (72.27) | 1,968 (39.26) | 1,117 (16.65) | 709 (5.68) |  |  |  |  |
| SCHIP |  |  |  |  |  | < 0.01 |  |  |  |
| Yes | 2,518 (8.18) | 1,333 (19.07) | 602 (15.98) | 250 (5.42) | 122 (1.30) |  |  |  |  |
| No | 25,134 (91.82) | 4,485 (80.93) | 3,402 (84.02) | 4,767 (94.58) | 10,166 (98.70) |  |  |  |  |
| Other |  |  |  |  |  | < 0.01 |  |  |  |
| Yes | 4,601 (10.75) | 925 (9.76) | 792 (12.82) | 939 (11.82) | 1,548 (10.27) |  |  |  |  |
| No | 34,265 (89.25) | 6,917 (90.24) | 4,716 (87.18) | 6,081 (88.18) | 13,311 (89.73) |  |  |  |  |
| Not Insured |  |  |  |  |  | < 0.01 |  |  |  |
| Yes | 4,115 (11.63) | 1,487 (22.06) | 946 (19.09) | 743 (11.34) | 580 (4.60) |  |  |  |  |
| No | 34,666 (88.37) | 6,326 (77.94) | 4,552 (80.91) | 6,261 (88.66) | 14,268 (95.40) |  |  |  |  |
| **Maternal Education** |  |  |  |  |  | < 0.01 |  |  |  |
| = High School | 13,197 (45.18) | 4,718 (73.77) | 2,442 (57.97) | 2,215 (43.25) | 2,470 (26.07) |  |  |  |  |
| Some College | 10,685 (31.29) | 1,912 (20.38) | 1,770 (31.83) | 2,322 (37.35) | 3,767 (35.13) |  |  |  |  |
| = 4 Yr College Degree | 13,647 (23.53) | 932 (5.85) | 1,122 (10.20) | 2,270 (19.40) | 8,117 (38.80) |  |  |  |  |
| **Race** |  |  |  |  |  |  |  |  |  |
| White only | 30,788 (75.11) | 4,942 (55.60) | 4,202 (70.49) | 5,848 (80.85) | 12,976 (84.26) | < 0.01 |  |  |  |
| Black only | 4,043 (15.01) | 1,610 (22.43) | 655 (17.97) | 542 (11.75) | 837 (8.75) |  |  |  |  |
| Other | 3,820 (9.88) | 1,227 (15.98) | 629 (11.55) | 598 (7.40) | 993 (6.99) |  |  |  |  |
| **Ethnicity** |  |  |  |  |  | < 0.01 |  |  |  |
| Hispanic | 3,424 (11.51) | 1,227 (20.98) | 569 (13.96) | 497 (9.56) | 770 (6.31) |  |  |  |  |
| Other | 35,306 (88.49) | 6,579 (79.02) | 4,923 (86.04) | 6,502 (90.44) | 14,051 (93.69) |  |  |  |  |
| **Sex** |  |  |  |  |  | 0.49 |  |  |  |
| Male | 23,320 (59.78) | 4,706 (58.20) | 3,333 (60.10) | 4,277 (61.02) | 8,820 (59.60) |  |  |  |  |
| Female | 15,520 (40.22) | 3,133 (41.80) | 2,173 (39.90) | 2,738 (38.98) | 6,034 (40.40) |  |  |  |  |
| **Region** |  |  |  |  |  | < 0.01 |  |  |  |
| Northeast | 7,842 (22.32) | 1,126 (17.33) | 850 (16.30) | 1,124 (18.25) | 2,910 (17.75) |  |  |  |  |
| Midwest | 5,508 (14.69) | 1,644 (20.35) | 1,366 (24.01) | 1,965 (25.90) | 3,870 (25.23) |  |  |  |  |
| South | 7,020 (18.19) | 3,032 (43.73) | 1,795 (40.05) | 2,023 (34.16) | 4,583 (35.54) |  |  |  |  |
| West | 14,859 (44.80) | 2,040 (18.59) | 1,497 (19.64) | 1,908 (21.68) | 3,496 (21.48) |  |  |  |  |
|  |  |  |  |  |  |  |  |  |  |
| **Relation to Child** |  |  |  |  |  | < 0.01 |  |  |  |
| Mother | 32,071 (79.8) | 6,723 (83.05) | 4,700 (83.16) | 5,823 (79.66) | 11,855 (76.98) |  |  |  |  |
| Other | 6,788 (20.20) | 1,118 (16.95) | 807 (16.84) | 1,196 (20.34) | 3,003 (23.02) |  |  |  |  |
|  |  |  |  |  |  |  |  |  |  |
|  | **Mean** | **Mean** | **Mean** | **Mean** | **Mean** | **p** |  |  |  |
|  | **(SE)** | **(SE)** | **(SE)** | **(SE)** | **(SE)** |  |  |  |  |
| **Age** | 9.95 (0.07) | 9.19 (0.06) | 9.54 (0.14) | 10.01 (0.13) | 10.38 (0.07) | <0.01 |  |  |  |
| **Severity of Condition** | 4.22 (0.04) | 5.28 (0.06) | 4.62 (0.06) | 4.14 (0.05) | 3.61 (0.04) | < 0.01 |  |  |  |
| **Total adults** | 2.07 (0.01) | 1.94 (0.04) | 2.01 (0.02) | 2.13 (0.01) | 2.11 (0.01) | < 0.01 |  |  |  |
| **Total kids** | 2.14 (0.02) | 2.51 (0.04) | 2.31 (0.03) | 2.23 (0.03) | 1.90 (0.02) | < 0.01 |  |  |  |
| SE = standard error; p = p value; FPL = federal poverty level |  |  |  |  |  |  |  |  |  |
| Percents, means, and standard errors are based on weighted analysis |  |  |  |  |  |  |  |  |  |
|  |  |  |  |  |  |  |  |  |  |

| **Table 2. Demographics by SES Stratum and Access to Specialty Care** |  |  |  |  |  |  |  |  |  |  |  |  |  |  |  |  |
| --- | --- | --- | --- | --- | --- | --- | --- | --- | --- | --- | --- | --- | --- | --- | --- | --- |
|  | **< 133% of FPL** |  |  | **133 – 199% of FPL** |  |  | **200 – 299% of FPL** |  |  | **>= 300% of FPL** |  |  |  |  |  |  |
|  | **No** | **Yes** | **p** | **No** | **Yes** | **p** | **No** | **Yes** | **p** | **No** | **Yes** | **p** |  |  |  |  |
|  | **n (%)** | **n (%)** |  | **n (%)** | **n (%)** |  | **n (%)** | **n (%)** |  | **n (%)** | **n (%)** |  |  |  |  |  |
| **Ethnicity** |  |  | 0.3 |  |  | 0.06 |  |  | 0.87 |  |  | 0.26 |  |  |  |  |
| Hispanic | 81 (19.45) | 520 (24.07) |  | 27 (9.79) | 266 (15.25) |  | 21 (10.32) | 241 (9.75) |  | 15 (8.80) | 377 (5.73) |  |  |  |  |  |
| Other | 333 (80.55) | 2778 (75.94) |  | 212 (90.21) | 2299 (84.75) |  | 177 (89.68) | 3232 (90.25) |  | 251 (91.20) | 7729 (94.27) |  |  |  |  |  |
| **Insurance** |  |  |  |  |  |  |  |  |  |  |  |  |  |  |  |  |
| Medicaid |  |  | < 0.01 |  |  | 0.46 |  |  | 0.57 |  |  | 0.05 |  |  |  |  |
| Yes | 213 (45.22) | 2306 (72.22) |  | 55 (40.52) | 908 (36.52) |  | 36 (20.93) | 564 (18.05) |  | 27 (9.83) | 464 (6.09) |  |  |  |  |  |
| No | 207 (54.78) | 1004 (27.78) |  | 185 (59.48) | 1663 (63.48) |  | 163 (79.07) | 2924 (81.95) |  | 240 (90.17) | 7664 (93.91) |  |  |  |  |  |
| Private |  |  | 0.42 |  |  | < 0.01 |  |  | < 0.01 |  |  | < 0.01 |  |  |  |  |
| Yes | 99 (24.86) | 1036 (30.01) |  | 128 (41.78) | 1697 (62.68) |  | 144 (69.20) | 2969 (84.26) |  | 230 (78.88) | 7808 (95.15) |  |  |  |  |  |
| No | 321 (75.14) | 2274 (69.99) |  | 112 (58.22) | 874 (37.32) |  | 55 (30.80) | 519 (15.74) |  | 37 (21.12) | 320 (4.85) |  |  |  |  |  |
| SCHIP |  |  | 0.11 |  |  | 0.12 |  |  | 0.59 |  |  | 0.63 |  |  |  |  |
| Yes | 67 (15.08) | 599 (20.76) |  | 30 (22.47) | 269 (14.23) |  | 3 (3.50) | 132 (4.98) |  | 2 (1.69) | 74 (1.18) |  |  |  |  |  |
| No | 252 (84.92) | 1853 (79.24) |  | 150 (77.53) | 1575 (85.77) |  | 161 (96.50) | 2337 (95.02) |  | 194 (98.31) | 5492 (98.82) |  |  |  |  |  |
| Other |  |  | 0.28 |  |  | 0.83 |  |  | 0.92 |  |  | 0.79 |  |  |  |  |
| Yes | 48 (8.33) | 465 (12.15) |  | 32 (15.03) | 434 (16.26) |  | 28 (12.59) | 507 (12.94) |  | 24 (11.20) | 861 (10.05) |  |  |  |  |  |
| No | 372 (91.67) | 2845 (87.85) |  | 208 (84.97) | 2137 (83.74) |  | 171 (87.41) | 2981 (87.06) |  | 243 (88.80) | 7267 (89.95) |  |  |  |  |  |
| Not Insured |  |  | < 0.01 |  |  | < 0.01 |  |  | < 0.01 |  |  | < 0.01 |  |  |  |  |
| Yes | 184 (45.96) | 528 (18.45) |  | 100 (39.20) | 353 (14.91) |  | 47 (30.63) | 279 (8.82) |  | 36 (15.75) | 261 (3.52) |  |  |  |  |  |
| No | 232 (54.04) | 2775 (81.55) |  | 140 (60.80) | 2211 (85.09) |  | 149 (69.37) | 3202 (91.18) |  | 231 (84.25) | 7864 (96.78) |  |  |  |  |  |
| **Maternal Education** |  |  | 0.54 |  |  | 0.88 |  |  | 0.39 |  |  | < 0.01 |  |  |  |  |
| = High School | 220 (74.10) | 1,879 (71.74) |  | 97 (57.59) | 1,070 (55.47) |  | 52 (33.57) | 1,014 (39.14) |  | 48 (22.15) | 1,195 (23.00) |  |  |  |  |  |
| Some College | 126 (18.73) | 858 (21.63) |  | 75 (31.48) | 856 (33.61) |  | 71 (36.76) | 1,144 (38.89) |  | 83 (48.92) | 1,975 (33.99) |  |  |  |  |  |
| = 4 Yr College Degree | 59 (7.17) | 466 (6.63) |  | 63 (10.93) | 569 (10.92) |  | 72 (29.67) | 1,232 (21.97) |  | 125 (28.93) | 4,717 (41.35) |  |  |  |  |  |
| **Race** |  |  | 0.72 |  |  | 0.41 |  |  | 0.21 |  |  | 0.43 |  |  |  |  |
| White only | 254 (60.51) | 2,192 (60.44) |  | 191 (72.74) | 2,015 (72.48) |  | 148 (73.09) | 2,963 (81.42) |  | 228 (79.96) | 7,222 (86.10) |  |  |  |  |  |
| Black only | 78 (20.80) | 562 (23.02) |  | 25 (12.17) | 265 (15.96) |  | 22 (13.84) | 221 (9.78) |  | 19 (8.85) | 357 (7.10) |  |  |  |  |  |
| Other | 82 (18.70) | 531 (16.54) |  | 24 (15.09) | 282 (11.56) |  | 28 (13.07) | 287 (8.80) |  | 19 (11.19) | 522 (6.79) |  |  |  |  |  |
| **Region** |  |  | 0.02 |  |  | 0.09 |  |  | 0.39 |  |  | 0.14 |  |  |  |  |
| Northeast | 53 (14.58) | 510 (17.80) |  | 22 (10.13) | 423 (18.68) |  | 27 (14.09) | 593 (18.29) |  | 45 (19.24) | 1689 (18.64) |  |  |  |  |  |
| Midwest | 72 (13.86) | 722 (21.18) |  | 57 (21.19) | 642 (23.90) |  | 42 (20.66) | 1001 (27.55) |  | 64 (19.88) | 2135 (25.93) |  |  |  |  |  |
| South | 167 (43.65) | 1257 (43.57) |  | 81 (50.96) | 834 (39.13) |  | 58 (41.56) | 989 (33.54) |  | 81 (39.11) | 2453 (34.61) |  |  |  |  |  |
| West | 128 (27.91) | 821 (17.45) |  | 80 (17.73) | 672 (18.29) |  | 72 (23.69) | 905 (20.62) |  | 77 (21.77) | 1851 (20.81) |  |  |  |  |  |
| **Relation to Child** |  |  | 0.55 |  |  | 0.80 |  |  | 0.10 |  |  | 0.89 |  |  |  |  |
| Mother | 364 (86.12) | 2864 (84.64) |  | 211 (84.19) | 2230 (85.71) |  | 171 (73.63) | 2935 (82.55) |  | 219 (77.74) | 6604 (78.41) |  |  |  |  |  |
| Other | 55 (13.88) | 446 (15.36) |  | 29 (15.81) | 340 (14.29) |  | 28 (26.37) | 552 (17.45) |  | 48 (22.26) | 1524 (21.59) |  |  |  |  |  |
| **Sex** |  |  | 0.93 |  |  | 0.89 |  |  | 0.97 |  |  | 0.02 |  |  |  |  |
| Male | 264 (57.67) | 1964 (58.01) |  | 154 (58.83) | 1521 (57.99) |  | 125 (58.49) | 2065 (58.28) |  | 169 (71.46) | 4699 (58.65) |  |  |  |  |  |
| Female | 155 (42.33) | 1346 (41.99) |  | 86 (41.17) | 1049 (42.01) |  | 74 (41.51) | 1419 (41.72) |  | 98 (28.54) | 3427 (41.35) |  |  |  |  |  |
|  |  |  |  |  |  |  |  |  |  |  |  |  |  |  |  |  |
|  | **No** | **Yes** | **p** | **No** | **Yes** | **p** | **No** | **Yes** | **p** | **No** | **Yes** | **p** |  |  |  |  |
|  | **Mean (SE)** | **Mean (SE)** |  | **Mean (SE)** | **Mean (SE)** |  | **Mean (SE)** | **Mean (SE)** |  | **Mean (SE)** | **Mean (SE)** |  |  |  |  |  |
| **Age** | 10.66 (0.60) | 8.80 (0.11) | < 0.01 | 9.96 (0.35) | 9.37 (0.24) | < 0.01 | 11.31 (0.41) | 9.77 (0.12) | < 0.01 | 10.84 (0.49) | 10.22 (0.08) | < 0.01 |  |  |  |  |
| **Severity of Condition** | 6.16 (0.22) | 5.65 (0.10) | 0.01 | 5.64 (0.27) | 5.17 (0.08) | < 0.01 | 5.77 (0.19) | 4.63 (0.08) | < 0.01 | 5.17 (0.18) | 3.93 (0.05) | < 0.01 |  |  |  |  |
| **Total adults** | 1.93 (0.10) | 1.96 (0.05) | 0.94 | 1.85 (0.09) | 2.04 (0.03) | 0.93 | 1.99 (0.06) | 2.12 (0.02) | 0.12 | 2.06 (0.05) | 2.10 (0.01) | 0.60 |  |  |  |  |
| **Total kids** | 2.52 (0.07) | 2.42 (0.04) | 0.90 | 2.27 (0.08) | 2.29 (0.05) | 0.03 | 2.11 (0.08) | 2.20 (0.04) | 0.26 | 1.97 (0.08) | 1.90 (0.02) | 0.38 |  |  |  |  |
| SE = standard error; p = p value; FPL = federal poverty level |  |  |  |  |  |  |  |  |  |  |  |  |  |  |  |  |
| Percents, means, and standard errors are based on weighted analysis |  |  |  |  |  |  |  |  |  |  |  |  |  |  |  |  |
|  |  |  |  |  |  |  |  |  |  |  |  |  |  |  |  |  |

| **Table 3. Mulitple Logistic Regression for Each SES Stratum** |  |  |  |  |  |  |  |  |  |  |  |  |  |  |  |
| --- | --- | --- | --- | --- | --- | --- | --- | --- | --- | --- | --- | --- | --- | --- | --- |
|  | **< 133% of FPL** |  |  |  |  |  | **133 – 199% of FPL** |  |  |  |  |  |  |  |  |
|  | **S ß** | **SE** | **p** | **OR** | **95% CI** |  | **S ß** | **SE** | **p** | **OR** | **95% CI** |  |  |  |  |
| **Age** | -3.68 | 0.06 | 0.12 | 0.91 | 0.82, 1.02 |  | 0.82 | 0.06 | 0.73 | 1.02 | 0.90, 1.16 |  |  |  |  |
| **Severity of Condition** | 3.44 | 0.13 | 0.23 | 1.16 | 0.91, 1.49 |  | -0.13 | 0.22 | 0.98 | 0.99 | 0.65, 1.52 |  |  |  |  |
| **Sex** |  |  |  |  |  |  |  |  |  |  |  |  |  |  |  |
| Male | - | - | - | - | - |  | - | - | - | - | - |  |  |  |  |
| Female | 0.23 | 0.21 | 0.79 | 1.06 | 0.70, 1.59 |  | 1.21 | 0.31 | 0.35 | 1.34 | 0.73, 2.47 |  |  |  |  |
| **Race** |  |  |  |  |  |  |  |  |  |  |  |  |  |  |  |
| White | - | - | - | - | - |  | - | - | - | - | - |  |  |  |  |
| Black | 0.10 | 0.33 | 0.93 | 1.03 | 0.54, 1.97 |  | 2.06 | 0.35 | 0.05 | 1.97 | 1.00, 3.90 |  |  |  |  |
| Other | -1.00 | 0.54 | 0.58 | 0.74 | 0.26, 2.13 |  | -2.33 | 0.52 | 0.11 | 0.43 | 0.16, 1.20 |  |  |  |  |
| **Ethnicity** |  |  |  |  |  |  |  |  |  |  |  |  |  |  |  |
| Non-Hispanic | - | - | - | - | - |  | - | - | - | - | - |  |  |  |  |
| Hispanic | 8.99 | 0.79 | < 0.01 | 10.19 | 2.17, 47.95 |  | 0.25 | 1.23 | 0.95 | 1.08 | 0.10, 11.94 |  |  |  |  |
| **Maternal Education** |  |  |  |  |  |  |  |  |  |  |  |  |  |  |  |
| = 4 yr College Degree | - | - | - | - | - |  | - | - | - | - | - |  |  |  |  |
| Some College | 0.78 | 0.52 | 0.67 | 1.25 | 0.45, 3.48 |  | 0.82 | 0.41 | 0.61 | 1.23 | 0.55, 2.75 |  |  |  |  |
| = High School | -1.30 | 0.41 | 0.42 | 0.72 | 0.32, 1.60 |  | 0.43 | 0.38 | 0.79 | 1.11 | 0.53, 2.31 |  |  |  |  |
| **Insurance** |  |  |  |  |  |  |  |  |  |  |  |  |  |  |  |
| Private | - | - | - | - | - |  | - | - | - | - | - |  |  |  |  |
| Medicaid | 3.42 | 0.29 | < 0.01 | 2.28 | 1.29, 4.03 |  | -0.61 | 0.27 | 0.57 | 0.86 | 0.51, 1.46 |  |  |  |  |
| SCHIP | 1.08 | 0.31 | 0.32 | 1.37 | 0.74, 2.52 |  | -0.9 | 0.43 | 0.48 | 0.74 | 0.32, 1.71 |  |  |  |  |
| Uninsured | -2.71 | 0.97 | 0.46 | 0.49 | 0.07, 3.28 |  | -7.75 | 1.16 | 0.04 | 0.09 | 0.01, 0.86 |  |  |  |  |
| Other | 0.23 | 0.53 | 0.87 | 1.09 | 0.39, 3.07 |  | -0.27 | 0.55 | 0.88 | 0.92 | 0.31, 2.68 |  |  |  |  |
| **Region** |  |  |  |  |  |  |  |  |  |  |  |  |  |  |  |
| Northeast | - | - | - | - | - |  | - | - | - | - | - |  |  |  |  |
| Midwest | 2.88 | 0.52 | 0.05 | 2.81 | 1.02, 7.70 |  | -1.18 | 0.47 | 0.40 | 0.67 | 0.27, 1.70 |  |  |  |  |
| South | 4.65 | 0.52 | 0.04 | 2.93 | 1.07, 8.05 |  | -6.23 | 0.66 | 0.02 | 0.22 | 0.06, 0.81 |  |  |  |  |
| West | 4.71 | 0.45 | < 0.01 | 3.56 | 1.48, 8.59 |  | -5.92 | 0.83 | 0.04 | 0.19 | 0.04, 0.95 |  |  |  |  |
| **Relation to Child** |  |  |  |  |  |  |  |  |  |  |  |  |  |  |  |
| Other | - | - | - | - | - |  | - | - | - | - | - |  |  |  |  |
| Mother | -0.56 | 0.43 | 0.65 | 0.82 | 0.35, 1.92 |  | 0.45 | 0.59 | 0.79 | 1.17 | 0.37, 3.73 |  |  |  |  |
| **Total Adults** | 1.93 | 0.13 | 0.06 | 1.29 | 0.99, 1.67 |  | 6.28 | 0.28 | < 0.01 | 2.73 | 1.57, 4.73 |  |  |  |  |
| **Total Kids** | -1.92 | 0.08 | 0.05 | 0.85 | 0.72, 1.00 |  | -0.63 | 0.11 | 0.57 | 0.94 | 0.75, 1.17 |  |  |  |  |
| **Age * Severity** | 1.10 | 0.01 | 0.64 | 1.00 | 0.99, 1.02 |  | -4.26 | 0.01 | 0.17 | 0.99 | 0.97, 1.01 |  |  |  |  |
| **Region * Severity** | -7.44 | 0.04 | 0.02 | 0.91 | 0.84, 0.99 |  | 0.83 | 0.06 | 0.85 | 1.01 | 0.90, 1.31 |  |  |  |  |
| **Uninsured * Severity** | -0.80 | 0.05 | 0.52 | 0.97 | 0.87, 1.07 |  | 1.73 | 0.10 | 0.40 | 1.09 | 0.89, 1.34 |  |  |  |  |
| **Uninsured * Race** | -0.73 | 0.39 | 0.80 | 0.91 | 0.42, 1.96 |  | -0.55 | 0.40 | 0.78 | 0.89 | 0.41, 1.97 |  |  |  |  |
| **Ethnicity * Region** | -5.72 | 0.22 | 0.04 | 0.64 | 0.42, 0.98 |  | 2.73 | 0.40 | 0.51 | 1.30 | 0.59, 2.87 |  |  |  |  |
| **Uninsured * Region** | -0.18 | 0.23 | 0.95 | 0.99 | 0.63, 1.53 |  | 2.33 | 0.26 | 0.35 | 1.28 | 0.77, 2.13 |  |  |  |  |
|  |  |  |  |  |  |  |  |  |  |  |  |  |  |  |  |
|  | **200 – 299% of FPL** |  |  |  |  |  | **= 300% of FPL** |  |  |  |  |  |  |  |  |
|  | **S ß** | **SE** | **p** | **OR** | **95% CI** |  | **S ß** | **SE** | **p** | **OR** | **95% CI** |  |  |  |  |
| **Age** | -2.52 | 0.07 | 0.31 | 0.94 | 0.82, 1.07 |  | 2.95 | 0.03 | 0.03 | 1.07 | 1.01, 1.14 |  |  |  |  |
| **Severity of Condition** | -7.61 | 0.18 | 0.05 | 0.71 | 0.50, 1.00 |  | -0.17 | 0.11 | 0.94 | 0.99 | 0.80, 1.23 |  |  |  |  |
| **Sex** |  |  |  |  |  |  |  |  |  |  |  |  |  |  |  |
| Male | - | - | - | - | - |  | - | - | - | - | - |  |  |  |  |
| Female | 0.05 | 0.26 | 0.97 | 1.01 | 0.61, 1.68 |  | 2.28 | 0.32 | 0.11 | 1.67 | 0.90, 3.12 |  |  |  |  |
| **Race** |  |  |  |  |  |  |  |  |  |  |  |  |  |  |  |
| White | - | - | - | - | - |  | - | - | - | - | - |  |  |  |  |
| Black | -0.78 | 0.43 | 0.47 | 0.74 | 0.32, 1.70 |  | -1.15 | 0.31 | 0.12 | 0.62 | 0.33, 1.14 |  |  |  |  |
| Other | 0.32 | 0.44 | 0.77 | 1.14 | 0.48, 2.68 |  | -1.75 | 0.80 | 0.35 | 0.47 | 0.10, 2.28 |  |  |  |  |
| **Ethnicity** |  |  |  |  |  |  |  |  |  |  |  |  |  |  |  |
| Non-Hispanic | - | - | - | - | - |  | - | - | - | - | - |  |  |  |  |
| Hispanic | 11.78 | 3.96 | 0.25 | 93.32 | 0.04, 1000 |  | 2.87 | 1.91 | 0.49 | 3.69 | 0.09, 155.55 |  |  |  |  |
| **Maternal Education** |  |  |  |  |  |  |  |  |  |  |  |  |  |  |  |
| = 4 yr College Degree | - | - | - | - | - |  | - | - | - | - | - |  |  |  |  |
| Some College | 2.75 | 0.41 | 0.09 | 1.99 | 0.90, 4.40 |  | -3.17 | 0.21 | < 0.01 | 0.48 | 0.32, 0.73 |  |  |  |  |
| = High School | 3.32 | 0.38 | 0.03 | 2.28 | 1.08, 4.84 |  | -0.89 | 0.26 | 0.37 | 0.79 | 0.47, 1.32 |  |  |  |  |
| **Insurance** |  |  |  |  |  |  |  |  |  |  |  |  |  |  |  |
| Private | - | - | - | - | - |  | - | - | - | - | - |  |  |  |  |
| Medicaid | -0.65 | 0.39 | 0.58 | 0.81 | 0.38, 1.73 |  | 0.03 | 0.33 | 0.97 | 1.01 | 0.53, 1.95 |  |  |  |  |
| SCHIP | 2.10 | 0.74 | 0.10 | 3.35 | 0.78, 14.33 |  | -0.41 | 0.92 | 0.65 | 0.66 | 0.11, 3.96 |  |  |  |  |
| Uninsured | -1.85 | 1.46 | 0.59 | 0.46 | 0.03, 8.10 |  | -4.36 | 1.32 | 0.07 | 0.09 | 0.01, 1.23 |  |  |  |  |
| Other | -0.46 | 0.35 | 0.64 | 0.85 | 0.43, 1.70 |  | -0.36 | 0.48 | 0.78 | 0.88 | 0.35, 2.24 |  |  |  |  |
| **Region** |  |  |  |  |  |  |  |  |  |  |  |  |  |  |  |
| Northeast | - | - | - | - | - |  | - | - | - | - | - |  |  |  |  |
| Midwest | -3.11 | 0.68 | 0.15 | 0.38 | 0.10, 1.43 |  | 2.04 | 0.18 | < 0.01 | 1.78 | 1.25, 2.54 |  |  |  |  |
| South | -5.49 | 0.91 | 0.13 | 0.25 | 0.04, 1.49 |  | 1.43 | 0.32 | 0.30 | 1.39 | 0.74, 2.59 |  |  |  |  |
| West | -5.87 | 1.12 | 0.15 | 0.20 | 0.02, 1.81 |  | 2.65 | 0.52 | 0.20 | 1.94 | 0.70, 5.40 |  |  |  |  |
| **Relation to Child** |  |  |  |  |  |  |  |  |  |  |  |  |  |  |  |
| Other | - | - | - | - | - |  | - | - | - | - | - |  |  |  |  |
| Mother | 2.34 | 0.41 | 0.05 | 2.20 | 0.99, 4.88 |  | 0.62 | 0.35 | 0.61 | 1.19 | 0.61, 2.35 |  |  |  |  |
| **Total Adults** | 0.79 | 0.14 | 0.32 | 1.15 | 0.87, 1.53 |  | 0.01 | 0.20 | 0.99 | 1.00 | 0.67, 1.49 |  |  |  |  |
| **Total Kids** | -0.32 | 0.10 | 0.73 | 0.97 | 0.79, 1.18 |  | -1.26 | 0.16 | 0.33 | 0.85 | 0.62, 1.17 |  |  |  |  |
| **Age * Severity** | -1.06 | 0.01 | 0.70 | 1.00 | 0.98, 1.02 |  | -5.57 | 0.01 | < 0.01 | 0.98 | 0.97, 0.99 |  |  |  |  |
| **Region * Severity** | 5.73 | 0.06 | 0.21 | 1.08 | 0.96, 1.21 |  | 0.27 | 0.02 | 0.87 | 1.00 | 0.96, 1.05 |  |  |  |  |
| **Uninsured * Severity** | 0.68 | 0.20 | 0.79 | 1.05 | 0.72, 1.54 |  | 1.54 | 0.06 | 0.01 | 1.19 | 1.05, 1.34 |  |  |  |  |
| **Uninsured * Race** | -3.81 | 0.44 | 0.01 | 0.34 | 0.14, 0.81 |  | 3.34 | 0.69 | 0.05 | 3.81 | 0.99, 14.63 |  |  |  |  |
| **Ethnicity * Region** | -9.69 | 1.10 | 0.31 | 0.33 | 0.04, 2.81 |  | -4.08 | 0.48 | 0.25 | 0.58 | 0.23, 1.48 |  |  |  |  |
| **Uninsured * Region** | 0.17 | 0.24 | 0.92 | 1.03 | 0.64, 1.65 |  | -3.18 | 0.28 | 0.03 | 0.55 | 0.32, 0.95 |  |  |  |  |
| Sß = standardized beta; SE = standard error; p = p value; FPL = federal poverty level |  |  |  |  |  |  |  |  |  |  |  |  |  |  |  |
| Regression models represent weighted analysis |  |  |  |  |  |  |  |  |  |  |  |  |  |  |  |
|  |  |  |  |  |  |  |  |  |  |  |  |  |  |  |  |
